# Voices in patients with schizophrenia talk in short, simple sentences

**DOI:** 10.1192/j.eurpsy.2025.714

**Published:** 2025-08-26

**Authors:** P. Del Olmo, P. Fuentes-Claramonte, J. Soler-Vidal, F. Neuhaus, L. López-Araquistain, L. Barbosa, P. Salgado-Pineda, S. Sarró, R. Salvador, J. Rosselló-Ximenes, P. J. McKenna, E. Pomarol-Clotet

**Affiliations:** 1 FIDMAG Germanes Hospitalàries Research Foundation; 2 CIBERSAM, ISCIII, Barcelona, Spain; 3 Maastricht University, Maastrich, Netherlands; 4 Hospital Sant Rafael; 5 Departament de Filologia Catalana i Lingüística General, Universitat de Barcelona, Barcelona, Spain

## Abstract

**Introduction:**

Auditory verbal hallucinations (AVH) are prevalent in schizophrenia and are often distressing. However, relatively little is known about their linguistic structure, although a number of authors have commented that they tend take the form of short, syntactically simple sentences (Tovar *et al* Schizophr Res 2019; 206 111-117; Corona-Hernández *et al* Schizophr Res 2022; 241 210-217). It has been suggested that these features may be related to the high frequency with which AVH feature insults and commands (which are normally short and simple).

**Objectives:**

We aimed to quantify sentence length and complexity of AVH in schizophrenia patients, and to examine how far length reductions were attributable to presence of insults and commands. We also examined the same variables in real speech from patients with schizophrenia and healthy controls.

**Methods:**

We transcribed verbatim AVH from 11 patients with very frequent AVH following a previously used protocol (Fuentes-Claramonte *et al* Sci Rep 2021; 23 18890). Mean sentence length and mean dependency distance (a measure of syntactic complexity) were calculated using the *udpipe* package in R. Insults and commands were also coded. For comparison, (real) speech samples were collected and transcribed from patients with schizophrenia (N=14) and healthy controls (N=15). All groups were matched for age, sex and estimated premorbid IQ.

**Results:**

We found that AVH sentences were on average significantly shorter (t_(37)_= -6.51, *p* < 0.001; see Fig. 1A) and syntactically simpler (t_(37)_= -4.37, *p* < 0.001; see Fig. 1B) than in the (real) speech of healthy controls. AVH sentences were also shorter (Fig. 1A) and simpler (Fig. 1B) than the speech of schizophrenia patients, although the latter comparison only approached significance (t_(37)_= -4.09, *p* < 0.001 and t_(37)_= -2.31, *p* = .08, respectively). After insults and commands were removed from the analysis, AVH sentences were still shorter (t_(37)_= -6.09, *p* < 0.001) and simpler (t_(37)_= -3.89, *p* < 0.001) than those in the speech of controls, and shorter (t_(37)_= -3.68, *p* < 0.01) than those in the speech of patients, but not simpler (t_(37)_= -1.86, *p* = 0.213).

**Image 1:**

**Figure:**
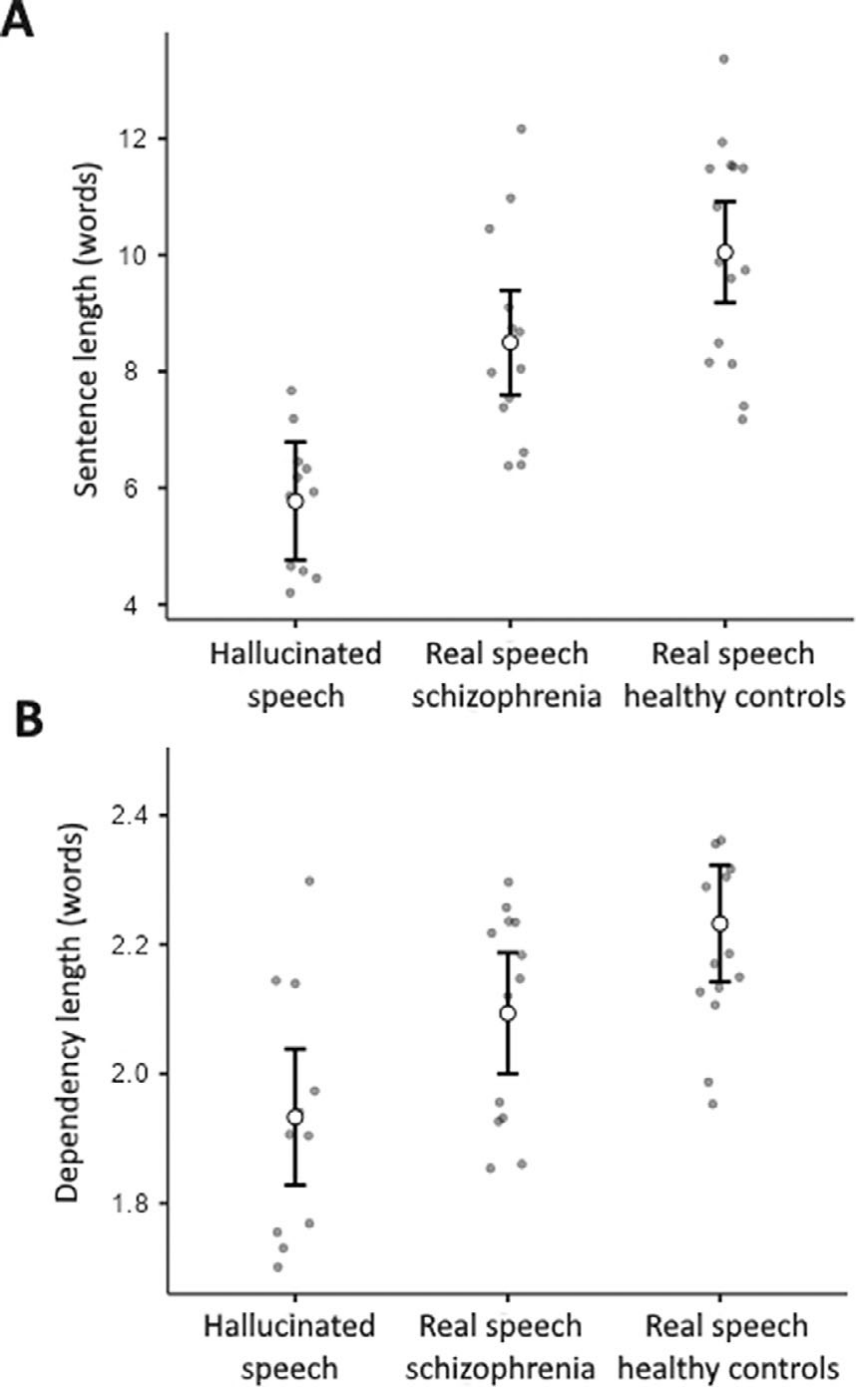

**Conclusions:**

From our data AVH mainly (though not exclusively) take the form of short and simple sentences. These features are not explained by presence of insults and commands.

**Disclosure of Interest:**

None Declared

